# Expansion of large granular lymphocytes after autologous hematopoietic stem cell transplantation

**DOI:** 10.1007/s12185-023-03540-y

**Published:** 2023-02-11

**Authors:** Mina Yoshida, Kensuke Matsuda, Kazuki Taoka, Akira Honda, Hiroaki Maki, Yosuke Masamoto, Masahiro Jona, Masako Nishikawa, Yutaka Yatomi, Mineo Kurokawa

**Affiliations:** 1 Department of Hematology and Oncology, Graduate School of Medicine, The University of Tokyo, 7-3-1 Hongo, Bunkyo-ku, Tokyo, 113-8655 Japan; 2 Department of Clinical Laboratory, The University of Tokyo Hospital, Tokyo, Japan; 3 Department of Cell Therapy and Transplantation Medicine, The University of Tokyo Hospital, Tokyo, Japan

**Keywords:** Large Granular Lymphocyte (LGL), Hematopoietic Stem Cell Transplantation, Autologous Transplantation, Malignant Lymphoma, Multiple Myeloma, Cytomegalovirus

## Abstract

Expansion of large granular lymphocytes (LGLs) is sometimes observed in allogeneic hematopoietic stem cell transplantation (HSCT) recipients, and is reported to be associated with a favorable transplant outcome. LGLs are also observed after autologous HSCT, but their clinical implications have not been well investigated. We retrospectively reviewed peripheral blood smears of consecutive autologous HSCT recipients. LGL lymphocytosis was defined as the observation of LGLs in the peripheral blood (> 20% white blood cells) in at least two consecutive blood tests. We evaluated the clinical impact of LGL lymphocytosis on autologous HSCT recipients. LGL lymphocytosis was observed in 18 of 197 patients (9.1%) who received autologous HSCT, at a median of 49 days after transplantation, with a median duration of 120.5 days. Incidence of cytomegalovirus reactivation was significantly higher in patients with LGL lymphocytosis than those without (16.7% vs. 3.3%, *p* = 0.038). No significant difference in survival rates was observed between groups (3 year OS 90.9% vs. 90.5%, *p* = 0.793 for lymphoma; 100 vs. 92.4%, *p* = 0.328 for myeloma). LGL lymphocytosis was observed in almost 10% of autologous HSCT recipients. In contrast to allogeneic HSCT, the duration of LGL was shorter and no significant improvement in survival was observed.

## Introduction

Large granular lymphocytes (LGLs) are characterized by an abundant cytoplasm with azurophilic granulation, composed of natural killer (NK) cells and cytotoxic T lymphocytes (CTL). LGLs have been reported to increase in various settings, including viral infection [1], during treatment with tyrosine kinase inhibitors (TKIs) [2], and after allogeneic hematopoietic stem cell transplantation (HSCT) [3, 4], which are thought to be caused by autoimmune processes, immune reactions to alloantigen stimulation, or over-compensatory CTL responses to infectious agents in humoral immunodeficiency. In allogeneic HSCT recipients, increased LGLs at post-transplantation are reportedly associated with cytomegalovirus (CMV) seropositivity, CMV reactivation, chronic graft-versus-host disease, and better prognosis [3, 5]. A previous report suggested that reactive LGLs had ligand receptors expressed by tumor cells and exerted an anti-tumor activity [6]. Furthermore, expansion of LGLs during treatment with dasatinib, a tyrosine kinase inhibitor, was significantly associated with better treatment response and occurrence of dasatinib-related adverse effects such as pleuritis and colitis, which are thought to be autoimmune reactivity of the expanded cytotoxic LGL cells [2].

In addition to these well-known causes of increased LGLs, LGL lymphocytosis was reported in various clinical settings in patients with hematologic malignancies [7, 8]. Among them, patients who underwent autologous HSCT showed a relatively frequent increase in LGLs; however, the clinical implications of LGL expansion post-autologous HSCT have not been well investigated. Previous studies have suggested that early absolute lymphocyte count recovery after autologous HSCT is associated with a better prognosis, and we hypothesized that it is partly because of the cytotoxic effect of increased LGLs [9, 10]. This study retrospectively reviewed the clinical characteristics of LGL expansion in patients who underwent autologous HSCT.

## Methods

We retrospectively reviewed the peripheral blood smear of consecutive patients who underwent their first autologous HSCT at the Department of Hematology & Oncology of the University of Tokyo Hospital between January 2007 and April 2021. LGL lymphocytosis was defined as the predominance of large lymphocytes containing typical azurophilic granules with a reniform or round nucleus with mature chromatin in the peripheral blood (> 20% WBCs) at least two consecutive blood tests. Some samples were subjected to immunophenotyping by flow cytometry. LGLs with CD3 + , CD8 + , and CD57 + phenotypes were defined as T-LGLs, whereas those with CD3–CD16 + and CD56 + phenotypes were defined as NK-LGLs [11].

The day of stem cell infusion was defined as day 0, and overall survival (OS) and progression-free survival (PFS) were defined to be calculated from day 0. We analyzed the association between LGL appearance and patients’ clinical characteristics (age, gender, disease type, stage, disease status before transplantation, detected chromosomal abnormalities, IPI (international prognostic index) for aggressive lymphoma, ISS (international staging system) for myeloma, HCT-CI (hematopoietic cell transplantation-specific comorbidity index), CMV serostatus, infused CD34 + cell dose, febrile neutropenia, viral infection) using Fisher’s exact test and Student’s t-test, whereas OS and PFS probabilities were compared using the log-rank test. CMV reactivation was defined as the detection of three or more CMV pp65 antigen-positive cells on two glass slides using CMV C10/C11 assay after autologous HSCT in patients who CMV IgG positivity had been confirmed before transplantation. Febrile neutropenia was defined as a condition marked by fever (axillary temperature 37.5℃ or higher) during a period of neutropenia (< 500/µL) after autologous HSCT. To eliminate immortal bias, landmark analysis was performed; LGL expansions were defined as the appearance of LGLs within 100 days from transplantation, and patients who died within 100 days from transplantation were excluded from the analysis. PFS was analyzed excluding patients whose disease progressed within 100 days after transplantation. Only the first autologous HSCT was included and was censored when the patient received the 2nd autologous HSCT. However, in the case of tandem transplantation for MM, the 2nd transplantation had been planned in advance and was performed not for relapse, and was not censored in the analysis. A *p* value of < 0.05 was considered statistically significant. EZR (Saitama Medical Center, Jichi medical university, Saitama, Japan, version 1.54) was used for statistical analysis [12]. The study was conducted following the principles of the Helsinki Declaration and was approved by the University of Tokyo Hospital Ethics Committee.

## Results

A total of 197 patients who received their first autologous HSCT and survived > 100 days from transplantation were identified. The median follow-up was 4.7 years (range, 3.5 months to 14.5 years), and 3-year OS and PFS were 90.8% (95% confidence interval CI 85.4–94.3%) and 74.7% (95% CI 67.4–80.5%), respectively. Diseases included 113 aggressive lymphomas, 23 indolent lymphomas, 7 Hodgkin lymphomas, 48 multiple myelomas, 4 acute promyelocytic leukemias and 2 POEMS (Polyneuropathy, Organomegaly, Endocrinopathy, Monoclonal protein, Skin changes) syndromes. Ten MM and one DLBCL patient underwent the 2nd autologous HSCT; among MM patients, 4 were tandem transplantation.

Eighteen patients (9.1%) were found to have persistent LGLs in the peripheral blood within 100 days of transplantation. Their diseases included nine diffuse large B-cell lymphomas (DLBCL), two mantle cell lymphomas, one follicular lymphoma, one Hodgkin lymphoma and five multiple myelomas. Between cases with and without LGLs, there was no significant difference in the ratio of lymphoma to MM and aggressive to indolent lymphoma, or whether the objective of transplantation was upfront consolidation or salvage at relapse. LGL expansion was observed in none of the four patients who underwent tandem transplantation for MM. LGLs appeared at a median of 49 (range 20–93) days post-transplantation, and all patients showed LGL expansion after engraftment (median, 11; range, 8–35 days). The median duration of LGL appearance was 120.5 (range 3–1373) days. Baseline characteristics between autologous HSCT recipients are shown in Table 1. Days before neutrophil engraftment after autologous HSCT were not significantly different between cases with and without LGL lymphocytosis (average 10.7 vs. 11.4 days, *p* = 0.392). LGL expansion was significantly correlated with the high or high-intermediate risk of the international prognostic index (IPI) in lymphoma (83.3 vs. 36.1%, *p* = 0.0034). CMV reactivation was checked in 135 out of 197 patients and observed at a median of 30.1 (range 10–37) days from transplantation in the overall cohort. CMV reactivation post-transplantation was significantly frequently observed in patients with LGLs (16.7 vs. 3.3%, *p* = 0.038). Among them, one and two cases of CMV colitis were observed in patients with and without LGLs, respectively. Febrile neutropenia occurred at a median of 5 (range 0–26) days from transplantation. Although there was no significant difference, the incidence of febrile neutropenia tended to be frequently observed in patients with LGLs (72.2 vs. 48.6%, *p* = 0.082). Notably, all these outcomes including febrile neutropenia and CMV reactivation were observed before the LGL appearance. The median periods from CMV reactivation and FN to LGL appearance were 7 (range 0–14) days and 42.5 (range 16–91) days, respectively. Eleven patients among 48 MM cases had started lenalidomide maintenance therapy within 100 days after autologous HSCT, and LGL lymphocytosis was observed in two of them (18%). One patient developed treatment-refractory LGL leukemia 3.8 years after autologous HSCT for DLBCL. The LGL phenotypes were determined by flow cytometry of the peripheral blood in six patients: three with T-cell phenotypes and three with the coexistence of T- and NK-cell phenotypes.

**Table 1:** Patient characteristics stratified by LGL appearance post-autologous HSCT

	Overall (*n* = 197, 100%)	LGL (*n* = 18, 9%)	No LGL (*n* = 179, 91%)	*P* value
Overall Patients				
Sex (F/M)	66/131 (34%/66%)	5/13 (28%/62%)	61/118 (34%/66%)	0.808
Age (years, average)	59 (range 19–89)	64 (40–70)	58 (19–89)	0.0678
Diseases				
Myeloma/Lymphoma	48/143	5/13	43/130	0.779
Others	6	0	6	
HCT-CI (average)	0.83	1.00	0.82	0.565
CMV serostatus				
CMV IgG +	154 (78.2%)	15 (83.3%)	139 (77.7%)	1.000
CMV reactivation +	9 (4.6%)	3 (16.7%) (Antigenemia: 2, Colitis: 1)	6 (3.3%) (Antigenemia: 4, Colitis: 2)	0.0382
Cell dose				
CD34 + cells (*10^6/kg, median)	3.50	3.55	3.50	0.642
Infection				
Febrile neutropenia	100 (50.8%)	13 (72.2%)	87 (48.6%)	0.0818
Viral infection +	11 (5.6%)	3 (CMV, 16.7%)	8 (4.5%) (CMV: 6, VZV: 2)	0.0666
Malignant lymphoma	*n* = 143	*n* = 13	*n* = 130	
IPI				
Low/Low-I/High-I/High	40/40/36/18	1/1/7/3	39/39/29/15	0.00341
Ann Arbor Stage				
I/II/III/IV	9/17/34/80	0/1/2/10	9/16/32/70	0.670
Response before transplantation				
CR/PR/SD-PD	106/25/11	11/1/1	95/24/10	0.688
Transplantation status				
Upfront/Relapse	60/79	6/7	54/72	1.000
Chromosomal abnormalities				
Positive/Negative	30/83 (26.5%)	4/3 (57.1%)	26/80 (24.5%)	0.228
Multiple myeloma	*n* = 48	*n* = 5	*n* = 43	
ISS				
I/II/III	17/19/8	1/1/2	16/18/6	0.289
D/S Stage (Myeloma)				
I/II/III	8/11/28	0/3/2	8/8/26	0.133
Response before transplantation				
CR/VGPR/PR/SD-PD	7/21/15/4	1/2/2/0	6/19/13/4	1.000
Chromosomal abnormalities				
Positive/Negative	11/31 (26.1%)	2/3 (40%)	9/28 (24.3%)	0.593

*LGL* large granular lymphocyte, *HCT-CI* hematopoietic cell transplantation-comorbidity index, *CMV*, cytomegalovirus, *IPI* international prognostic index, *CR* complete response, *PR* partial response, *SD* stable disease *PD* progressive disease, *ISS* international staging system, *D/S* Durie/Salmon, *VGPR* very good partial response

**Fig. 1:**
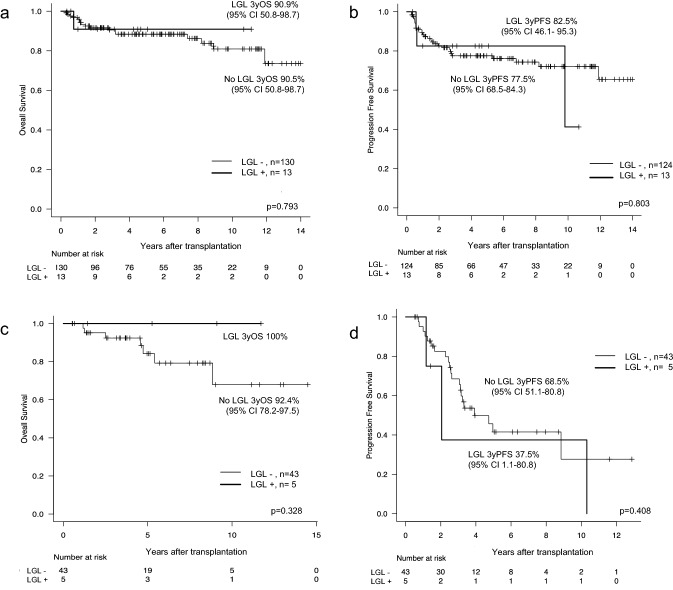
Outcomes after autologous stem cell transplantation. **a**. Overall survival for patients with lymphoma, **b**. Progression-free survival for patients with lymphoma, **c**. Overall survival for patients with myeloma, **d**. Progression-free survival for patients with myeloma

The outcomes are shown in Fig. 1. In patients with malignant lymphoma, no difference was observed in OS and PFS between patients with LGL expansion and the control (3-year OS 90.9 vs. 90.5%, *p* = 0.793; 3-year PFS 82.5 vs. 77.5%, *p* = 0.803, respectively). Similarly, in patients with multiple myeloma, no significant differences in OS and PFS were observed between patients with LGL expansion and the control (3-year OS 100 vs. 92.4%, *p* = 0.328; 3-year PFS 37.5 vs. 68.5%, *p* = 0.408, respectively).

## Discussion

This is the first study to explore the clinical features and prognoses of patients with LGL lymphocytosis post-autologous HSCT. LGL lymphocytosis was confirmed in 9.1% of patients undergoing autologous HSCT, and CMV reactivation and febrile neutropenia were more frequently detected in the cases with LGLs. The effect of LGL appearance on the prognosis of hematologic malignancies after autologous HSCT was not observed.

The time to LGL appearance and its duration were both shorter than those of allogeneic HSCT [5], which might be the reason that LGL lymphocytosis has not been paid much attention. Continuous alloantigen stimulation may cause durable LGL lymphocytosis in allogeneic HSCT settings, which does not exist in autologous HSCT. Although most cases with LGL expansion resolved spontaneously, one of the patients progressed to treatment-resistant LGL leukemia. Only a few cases of LGL leukemia after autologous HSCT have been reported, and it is presumably caused by chronic excess immune stimulation due to persistent infection, autoimmunity, or a malignancy-associated antigen in tumor immune surveillance; however, the specific event preceding the development of LGL leukemia has not yet been identified [13–15]. Chronic LGL lymphocytosis should be carefully followed, and if it persists for a long enough time, confirmation of clonality may be required.

As clinical outcomes, OS or PFS improvement was not observed in autologous HSCT recipients with LGL lymphocytosis in either malignant lymphoma or multiple myeloma, which was inconsistent with allogeneic HSCT recipients. One possible reason may be that the shorter duration of LGL lymphocytosis than in allogeneic HSCT might have attenuated its impact on disease prognosis. Another reason may be the difference in antigen stimulation. In contrast to allogeneic HSCT, there was no alloantigen in autologous HSCT. It was possible that alloantigen stimulation was necessary for expanded LGLs to exert the anti-tumor effect.

Notably, among patients with malignant lymphoma, the proportion of patients with LGL expansion was significantly higher in patients with higher IPI, which suggested a population with a poor prognosis. Considering the similar prognosis with or without LGLs in patients with malignant lymphoma, increased LGLs may have potentially improved the prognosis in malignant lymphoma. Further large-scale studies were needed to confirm this notion. The possible mechanism for the correlation between higher IPI and LGL lymphocytosis may be that patients with higher IPI tend to be older and with advanced stages based on its scoring system, which are also risk factors for infection [16]. Therefore, patients with higher IPI might be more vulnerable to apparent or subclinical infections after autologous HSCT, which may lead to NK and cytotoxic T-cell appearance [1]. The finding that LGL expansion was frequently observed after various infections in this study may support this hypothesis.

As a mechanism for the LGL appearance, immune stimulation by CMV reactivation was estimated. Furthermore, the fact that febrile neutropenia was more frequently observed before the appearance of LGLs suggests that evident infections and some other undiagnosed subclinical infections stimulate the cytotoxic immune reaction in those immunosuppressed hosts, which leads to LGL appearance. Additionally, in our institution, evaluation of CMV reactivation is not routinely performed, but frequently monitored especially in cases with undetermined fever. Because of this, the expansion of LGLs after CMV reactivation may partially be affected by other undetermined infections. Although alloantigen stimulation does not exist unlike in allogeneic HSCT, it is suspected that immune reconstruction after autologous HSCT might stimulate LGL expansion, which should be investigated further in the future.

This study has some limitations. First, phenotypes and clonality of LGLs were evaluated in a limited number of patients, and the immunological characteristics remain unclear. Moreover, Because the reconstruction of NK cells and CD8 + T cells, which constitute LGLs, is reported usually earlier than that of CD4 + T cells and B cells [17], the effect of LGL lymphocytosis on the reconstitution of other cell lineages should be investigated further in the future. Secondly, this study is a single-center, retrospective study and the number of patients with LGL lymphocytosis was restricted, therefore the impact of LGL lymphocytosis on OS and PFS may be difficult to be fully evaluated in this study. However, it is because detailed morphological assessments of LGLs in peripheral blood are not routinely conducted in every institute. Third, because the transplantation outcome is not significantly different between cases with and without LGLs partly because of the limited sample size, practical clinical meanings of LGLs are still mostly unclear. It should be further investigated in the future to clarify its clinical indication.

In conclusion, LGL lymphocytosis was confirmed in 9.1% of patients undergoing autologous HSCT. In contrast to allogeneic HSCT recipients or patients during TKI therapy, LGL expansion was not significantly associated with preferable outcomes in autologous HSCT recipients.

## Data Availability

The datasets generated and analysed during the current study are not publicly available due to ethical restrictions but are available from the corresponding author on reasonable request.

## References

[1] Rossi D, Franceschetti S, Capello D, De Paoli L, Lunghi M, Conconi A (2007). Transient monoclonal expansion of CD8+/CD57+ T-cell large granular lymphocytes after primary cytomegalovirus infection. Am J Hematol.

[2] Mustjoki S, Ekblom M, Arstila TP, Dybedal I, Epling-Burnette PK, Guilhot F (2009). Clonal expansion of T/NK-cells during tyrosine kinase inhibitor dasatinib therapy. Leukemia.

[3] Nann-Rütti S, Tzankov A, Cantoni N, Halter J, Heim D, Tsakiris D (2012). Large granular lymphocyte expansion after allogeneic hematopoietic stem cell transplant is associated with a cytomegalovirus reactivation and shows an indolent outcome. Biol Blood Marrow Transplant.

[4] Mohty M, Faucher C, Vey N, Chabannon C, Sainty D, Arnoulet C (2002). Features of large granular lymphocytes (LGL) expansion following allogeneic stem cell transplantation: a long-term analysis. Leukemia.

[5] Kim D, Al-Dawsari G, Chang H, Panzarella T, Gupta V, Kuruvilla J (2013). Large granular lymphocytosis and its impact on long-term clinical outcomes following allo-SCT. Bone Marrow Transplant.

[6] Costello R, Sivori S, Mallet F, Sainty D, Arnoulet C, Reviron D (2002). A novel mechanism of antitumor response involving the expansion of CD3+/CD56+ large granular lymphocytes triggered by a tumor-expressed activating ligand. Leukemia.

[7] Matsuda K, Taoka K, Jona M, Masuda A, Arai S, Nakamura F (2017). Clinical features of hematological disorders with increased large granular lymphocytes (LGLs): a retrospective study. Ann Hematol.

[8] Wolniak KL, Goolsby CL, Chen Y-H, Chenn A, Singhal S, Mehta J (2013). Expansion of a clonal CD8+CD57+ large granular lymphocyte population after autologous stem cell transplant in multiple myeloma. Am J Clin Pathol.

[9] Porrata LF, Gertz MA, Inwards DJ, Litzow MR, Lacy MQ, Tefferi A (2001). Early lymphocyte recovery predicts superior survival after autologous hematopoietic stem cell transplantation in multiple myeloma or non-Hodgkin lymphoma. Blood.

[10] Joao C, Porrata LF, Inwards DJ, Ansell SM, Micallef IN, Johnston PB (2006). Early lymphocyte recovery after autologous stem cell transplantation predicts superior survival in mantle-cell lymphoma. Bone Marrow Transplant.

[11] Watters RJ, Liu X, Loughran TP (2011). T-cell and natural killer-cell large granular lymphocyte leukemia neoplasias. Leuk Lymphoma.

[12] Kanda Y (2013). Investigation of the freely available easy-to-use software ‘EZR’ for medical statistics. Bone Marrow Transplant.

[13] Gill H, Ip AHW, Leung R, So JCC, Pang AWK, Tse E (2012). Indolent T-cell large granular lymphocyte leukaemia after haematopoietic SCT: a clinicopathologic and molecular analysis. Bone Marrow Transplant.

[14] Narumi H, Kojima K, Matsuo Y, Shikata H, Sekiya K, Niiya T (2004). T-cell large granular lymphocytic leukemia occurring after autologous peripheral blood stem cell transplantation. Bone Marrow Transplant.

[15] Dearden C (2011). Large granular lymphocytic leukaemia pathogenesis and management. Br J Haematol.

[16] Eyre TA, Wilson W, Kirkwood AA, Wolf J, Hildyard C, Plaschkes H (2021). Infection-related morbidity and mortality among older patients with DLBCL treated with full- or attenuated-dose R-CHOP. Blood Adv.

[17] Auletta JJ, Lazarus HM (2005). Immune restoration following hematopoietic stem cell transplantation: an evolving target. Bone Marrow Transplant.

